# Behavioral Changes in Patients With Prader-Willi Syndrome Can Mask Severe Physical Illness

**DOI:** 10.1210/jcemcr/luac034

**Published:** 2023-02-03

**Authors:** Liselotte Van Loo, Annick Vogels, Anne Rochtus

**Affiliations:** Department of Pediatric Endocrinology, University Hospitals Leuven, 3000 Leuven, Belgium; Center for Human Genetics, University Hospitals Leuven, 3000 Leuven, Belgium; Department of Pediatric Endocrinology, University Hospitals Leuven, 3000 Leuven, Belgium; Department of Pediatric Neurology, University Hospitals Leuven, 3000 Leuven, Belgium

**Keywords:** Prader-Willi syndrome, behavioral changes, severe underlying illness

## Abstract

Behavioral and psychiatric problems are common in patients with Prader-Willi syndrome (PWS), while physical complaints such as pain, fever, and vomiting are rare due to a high pain threshold and dysregulation of temperature control. PWS patients have an increased mortality rate, some due to undiagnosed life-threatening diseases. We describe 2 patients with PWS whose behavioral changes, initially thought to be part of their behavioral phenotype, delayed the final diagnosis of a life-threatening underlying illness. A 13-year-old girl with PWS presented with a sudden change in behavior including aggression, scratching, and self-injury. She was seen by several health care providers, and after 5 months the diagnosis of pyosalpinx was made, for which laparoscopic resection of an infected tailgut cyst was performed, resolving the behavioral symptoms. A 38-year-old man with PWS presented with recurrent vague inguinal pain and nonepileptic seizures. After several years of consulting physicians and psychiatrists, including several hospital admissions, the diagnosis of bilateral inguinal hernia was made. After surgical correction, the pain and seizures ceased. In PWS patients presenting with unexplained behavioral changes and unusual somatic complaints, clinicians should perform an extensive clinical examination and consider underlying physical illness rather than attribute the problem to the behavioral phenotype.

Prader-Willi syndrome (PWS) is a neurodevelopmental disorder, caused by lack of expression of genes inherited from the paternal chromosome 15q11-q13 region. PWS results from a paternally inherited deletion of the 15q11.2-q13 region, from uniparental maternal disomy, an imprinting defect (ID), or a translocation involving chromosome 15. Patients with PWS have dysmorphic features (narrow bifrontal diameter, almond-shaped palpebral fissures, narrow nasal bridge), hypogenitalism, axial hypotonia, and feeding difficulties (from poorly controlled sucking) in the neonatal period, with a gradual progression toward excessive eating and insatiability. If untreated, PWS patients develop morbid obesity and the consequences of associated metabolic syndrome [1]. PWS is associated with mild to moderate cognitive impairment, behavioral problems such as temper tantrums, compulsivity, skin picking, and self-harming behavior, further progressing into psychiatric disorders including anxiety, mood disorders, and psychosis.

Furthermore, patients with PWS have altered pain tolerance and dysregulated temperature control. The changes in pain tolerance are attributed to an impaired peripheral somatosensory function and altered central processing of painful stimuli, possibly mediated by abnormal GABA-A receptor binding [1, 2]. These interoceptive disturbances seem to be associated with self-stimulating activities including skin picking and self-harm behavior [2, 3]. Dysfunction of the autonomous nervous system, more specifically diminished parasympathetic activity, is associated with faulty central control of thermoregulation in patients with PWS [4].

Together with the reduced occurrence of vomiting [5], it is no surprise that intra-abdominal pathology is a frequent cause of morbidity and mortality in patients with PWS [6]. Different case reports on intra-abdominal complications in patients with PWS, such as acute gastric dilation or gastric rupture have been published [7, 8]. They often present with vague and nonspecific symptoms, such as vomiting and abdominal pain [9] or loss of appetite.

We describe 2 patients with PWS, who consulted their health care provider because of behavioral changes and abdominal complaints initially attributed to the behavioral phenotype in PWS. The patients were ultimately diagnosed with severe intra-abdominal pathology for which surgical intervention was required. We urge caution if PWS patients present with newly onset abdominal symptoms or change in behavior, as these could be the only signs of life-threatening underlying illness.

## Case Presentation

We present 2 patients with PWS, presenting with atypical symptoms of an underlying physical illness.

**Figure 1.**
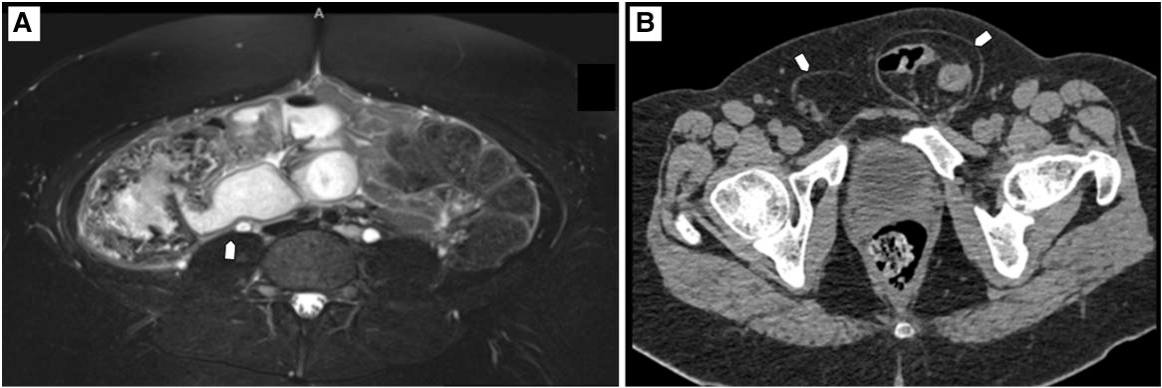
A, T2-weighted MRI scan showing an intra-abdominal mass and pyosalpinx in a 13-year-old girl with PWS. B, CT scan showing bilateral inguinal hernia in a 38-year-old male with PWS.

A 13-year-old girl with PWS confirmed by genetic testing (maternal uniparental disomy of the 15q11q13 region) and moderate intellectual disability (no formal IQ testing) presented in May 2021 to the pediatric outpatient clinic. She demonstrated constipation and soiling paired with a change in behavior described as increasing episodes of self-harm, skin picking, and aggression toward other children. She was seen by multiple physicians and treated with laxatives for multiple months. A genital physical examination was not performed because of her lack of cooperation. Five months later in October 2021, when vaginal soiling was reported by the parents, abdominal computed tomography (CT) and magnetic resonance imaging (MRI) scans were performed showing a pyosalpinx and an intra-abdominal mass (Fig. 1A). Blood sampling at this stage showed a marked increase in inflammatory markers (C-reactive protein 86 mg/L and sedimentation rate 62 mm/h). An explorative laparoscopy was performed and an infected tailgut cyst was diagnosed. After drainage of the abscess and antibiotic therapy, the constipation, soiling, and behavioral symptoms ceased.

A 38-year-old male with PWS confirmed by genetic testing (15q11-12 deletion) and mild intellectual disability (total IQ 56) was seen in 2015 with complaints of vague inguinal and abdominal pain, leg and back pain, “tingling in the hands,” and headaches during the night. The general practitioner suspected an inguinal hernia. The man was referred to a physiotherapist who attributed the pain to inflammation of the adductor muscles. X-rays of the hip and pelvis and MRI of the spine were normal. Physiotherapy did not resolve the symptoms and there was a further increase in abdominal and leg pain, as well as tingling in the hands. He was seen by an internist who diagnosed swollen lymph glands and he referred him to the urologist. Ultrasound examination was normal. Amitriptyline was prescribed as a treatment for depression and pain.

A first seizure occurred in 2016, described by the parents as “falling down, convulsions, growling, excessive salivation, grasping his head.” After the seizure he complained of muscle pain and headache. Electroencephalography (EEG) performed at the emergency department was normal. Corticosteroids were prescribed. In 2017, the seizures occurred more frequently and topiramate was prescribed. A repeat electroencephalography was normal. Polysomnography showed sleep apnea and he was started on continuous positive airway pressure (CPAP) therapy. Lormetazepam was prescribed for his anxiety. In January 2018, he had several severe and prolonged seizures episodes of reduced consciousness lasting more than 2 hours. After administration of diazepam at the emergency department, he was transferred to the intensive care unit. He was diagnosed with epilepsy and levetiracetam was prescribed by the neurologist. Because of severe behavioral problems and fugue behavior he was transferred to the isolation room, which induced a further increase in seizures and even more behavioral difficulties. During this admission, he was diagnosed with psychogenic nonepileptic seizures and transferred to a psychiatric inpatient unit for intellectually disabled persons with behavioral difficulties, where he was treated with eye movement desensitization and rehabilitation (EMDR) therapy for posttraumatic stress disorder.

In the following months, his condition worsened with increasing seizures, abdominal pain, urinary incontinence, and irritability. He was seen by a general practitioner, pneumologist, endocrinologist, general internist, and neurologist. Finally, an abdominal CT scan was performed, which revealed a bilateral inguinal hernia with enclosed omental and intestinal loops in 2021 (Fig. 1B), for which surgical reduction was performed. We could not recollect any information on inflammatory markers or lactate at the time of operation. After surgery, behavioral symptoms, nonepileptic seizures, and pain disappeared.

In both patients, there was a major discrepancy between the preexisting behavioral phenotype and the newly emerging complaints, there was no fever, and abdominal symptoms were only very vague. The patients could not adequately describe their complaints, and no comprehensive physical examination with genital inspection was performed, delaying the diagnosis of the underlying illness. Immediately after the resolution of the physical illness, the behavioral changes stopped in both patients, establishing the causal link between the genitoabdominal problems and the behavioral change.

## Discussion

Optimal management of patients with PWS involves both the management of several comorbidities that constitute the syndrome, as well as the ongoing assessment for the emergence of associated conditions and complications that evolve over the lifespan of these individuals. Butler et al [1] recently reported a concise overview of comorbidities and complications seen in patients with PWS, including life-threatening gastrointestinal problems, although genitoabdominal problems have not been included in this review.

We report 2 young adults with possible life-threatening genitoabdominal complications that remained undetected for a long time, presumably because of the atypical presentation. The girl was known with constipation, soiling, and a change in behavior. The man presented with longstanding vague inguinal pain, pain in the legs, general discomfort, behavioral changes, and nonepileptic seizures for multiple years. Only after repeated urging by the parents, additional physical and genital examinations were performed leading to the diagnosis of a genitoabdominal abscess in the girl and an inguinal hernia in the man. This late detection may be explained on one hand by the atypical symptoms, including vague abdominal complaints and a change in behavior, on the other hand by the difficult cooperation of PWS patients on physical examination.

It is well known that PWS individuals have a high pain threshold, impaired thermoregulation, and lack of vomiting, and may therefore not respond normally to abdominal infection or painful stimuli, with behavioral changes and vague physical complaints as the only clinical signs [2]. Severe abdominal problems, including gastric dilation due to binge eating without regurgitation, gastroparesis, gastric inflammation, gastrointestinal infections, and gastric necrosis with rupture have been reported [7-9]. These complications often present with mild symptoms, such as loss of appetite and mild abdominal pain, and often end in severe septic shock with a high mortality rate. Until now, only 11 cases have been reported (overview in Table 1). Most cases presented with vague or atypical symptoms, and 6/11 patients died.

**Table 1.** Reported cases of severe physical illness presenting with atypical symptoms in patients with Prader-Willi syndrome

Author/Date	Patient	Symptoms	Diagnosis	Treatment/outcome
Wharton RH et al (1997) [7]	36y, F, UPD	Fever, vomiting, abdominal pain	Gastric necrosis	Gastrectomy, cholecystectomy. Sepsis, ARDS, DIC, mechanical ventilation, recovery
	23y, F, del15q11-13	Abdominal pain, vomiting	Gastric dilation with necrosis	Death by sepsis and respiratory failure
	21y, F, UPD	Fever, short duration of abdominal pain	Stomach perforation, intra-abdominal abscess	Total parenteral nutrition, mechanical ventilation, tracheotomy, recovery
	37y, F, del15q11-13	Cardiac arrest, diarrhea	Postmortem: gastric dilation	Death
	7y, F, del15q11-13	Abdominal pain, distention, vomiting	Gastric dilation, food residue	Fasting, recovery
	6y, F, del15q11-13	Sudden abdominal swelling	Gastric dilation	Spontaneous resolution
Blat C et al 2017 [8]	5y, M	Abdominal dilation, hypovolemic shock	Abdominal compartment syndrome, gastric dilation	Admission to PICU, fluid resuscitation, fasting, recovery
Stevenson DA et al 2007 [9]	17y, M	Binge eating, abdominal pain, vomiting	Gastric necrosis and perforation	Gastrectomy. Death by sepsis and multisystem organ failure
	24y, M	Abdominal pain, vomiting	Gastric perforation and necrosis	Partial gastrectomy. Death by septic shock and multisystem organ failure
	22y, M	Weight loss, vomiting	Gastric rupture	Death
	49y, M	Ulcer	Gastric rupture	Death

If the molecular defect is not mentioned, it was not included in the case report.

Abbreviations: ARDS, acute respiratory distress syndrome; DIC, diffuse intravascular coagulopathy, F, female; M, male; PICU, Pediatric Intensive Care Unit; UPD, uniparental disomy; y, years.

In addition to the impaired somatosensory function, PWS patients have mild to moderate intellectual disability, possibly attributing to the difficulty expressing their health complaints. It has been proven that health problems are less likely to be diagnosed in patients with intellectual disability [10].

We conclude that the increased mortality rate seen in PWS patients [6] may partially be due to unrecognized or late detection of severe life-threatening illness. Several previous case reports reported PWS patients with gastric dilation and/or gastric necrosis leading to death. Hedgeman et al found that the incidence of all-cause mortality was significantly higher in the PWS population compared with the matched general population controls [6]. Among the 52 deaths were several sudden or unobserved deaths, raising the possibility that some of these fatalities of unknown etiology could be due to undiagnosed genitoabdominal problems.

Our 2 case reports emphasize the importance of a multidisciplinary approach to the care of PWS patients, including endocrinologists with an interest in behavior and child psychiatrists with interest in physical problems. There is a paucity of data supporting the use of medical approaches documented for PWS; however, standard recommendations for treatment have been published [1]. The multidisciplinary team should be alert for underlying physical illness, especially when the patients’ caretakers report abnormal behavior, and a thorough clinical examination including genital examination should be performed in every PWS patient presenting with an unexplained change in behavior and vague physical complaints. Parents and caretakers should be informed about the atypical presentation of possible life-threatening illnesses in PWS, and there should be a low threshold for seeking medical attention.

## Learning Points

- Behavioral problems, including temper tantrums, stubbornness, aggressive outbursts, and self-injuring behavior, as well as psychiatric comorbidities including autism spectrum disorder, anxiety, mood disorders, and psychosis are common in children and adults with Prader-Willi syndrome (PWS).
- Individuals with PWS have a high pain threshold, impaired temperature regulation, and a high threshold for vomiting leading to a risk of delay in the diagnosis of severe physical problems.
- PWS patients have difficulty expressing their health problems; therefore, information from caregivers and family can provide important clues in the diagnostic odyssey.
- A change in behavior can be the only presenting symptom of a serious physical illness in PWS patients and may be mistaken as part of their behavioral phenotype, causing diagnostic delay.
- Health care providers should be cautious about a possible underlying physical disease when a behavioral change occurs in patients with PWS.

## Data Availability

Data sharing is not applicable to this article as no datasets were generated or analyzed during the current study.

## Abbreviations

CT: computed tomography
MRI: magnetic resonance imaging
PWS: Prader-Willi syndrome
